# On the Pacinian Corpuscles on the Nerves of Men and Mammalia

**Published:** 1845-01

**Authors:** 


### ART. VIII.

*Ueber die Pacinischen Körperchen an den Nerven des Menschen und der Säugethiere.* Von J. HENLE und A. KÖLLIKER. Mit drei Tafeln.—*Zurich*, 1844.

*On the Pacinian Corpuscles on the Nerves of Men and Mammalia.* By J. HENLE and A. KÖLLIKER. With Three Plates.—*Zurich*, 1844. 4to, pp. 40.

ONE might have thought that all that could be seen with the naked eye in the anatomy of the human body had been described some time since; but it is not so. Here is a genuine discovery, and one which ought to have been made a century ago; for many of our readers will, when they read of them, remember that they, as well as ourselves, have seen, without observing, the Pacinian bodies a dozen times. The discovery of one's own heedlessness generates a humour in which remarks had better be suppressed; so we shall proceed straightway to the analysis of the work before us.

The name of these new bodies has been given to them by Henle and Kölliker in honour of their discoverer, Filippo Pacini, an Italian physician, who first noticed them in the beginning of 1830. They then appeared to him to be nothing more than little elliptical whitish masses of indurated cellular tissue attached to the nerves of the hand, and he took little notice of them. However, when he found them again and again, and on the nerves of the feet as well as of the hands, he began to think they meant something, and he undertook an extensive investigation of them. His first (and very imperfect) account was communicated in a letter to the 'Società Medico-Fisica di Firenze,' in October 1835, and was published in the March and April parts of the 'Nuovo Giornale dei Litterati,' (p. 109.) But before this, in 1833, the same bodies had been detected by A. G. Andral, Camus, and Lacroix, to whom the preparation of the nerves of the hand had been given as a subject in a *concours* for the place of assistant to the professor of anatomy; and in 1836,

Cruveilhier noticed the fact in his 'Anatomie Descriptive,' t. iv. p. 822; but he added that the little bodies, though gangliform, did not properly belong to the nerves, and were only weakly attached to them; and he regarded them as the consequences of external pressure, assigning for his opinion some imperfect erroneous observations. In 1837, they were described imperfectly by A. G. Andral, who considered them as ganglia of the nerves of tact; and in 1838, Blandin also noticed them in his 'Anatomie Descriptive,' t. ii. p. 675.

But thus far no real progress was made; and it was reserved for Pacini, who first observed these bodies, to gain also the honour of first giving a circumstantial account of them. He had indeed already described them more accurately than any of the French observers; but his next description, which he published in a separate work, entitled 'Nuovi Organi, scoperti nel corpo umano da Filippo Pacini di Pistoja,' 1840, left little to be added, except what could be learnt from a more exact microscopic dissection. This last has been added by Henle and Kölliker; and now it only remains to discover the true physiology of the Pacinian bodies, at which hitherto guesses only have been made.

Pacini's account, we have already said, was completely accurate, as far as it went; but for brevity's sake, we shall make our abstract from that given by Henle and Kölliker, by whom the description has been extended.

The Pacinian bodies are found not in man alone, but in all the domesticated mammalia, and probably in others also. In all they are connected with the nerves. In the human subject, from the first to the eighteenth year of life, they are invariably found in the cutaneous (but not in the muscular) nerves of the palm and sole. They have also been found, sparingly and inconstantly, in nerves at the wrist and elbow; in the upper arm, fore-arm, and thigh, and intercostal nerve, the sacral plexus, solar plexus, and the plexuses adjacent to it. In the mammalia they are also found, but less constantly in the soles; and in the cat they occur in great numbers in the branches of the sympathetic plexuses, in the mesentery and mesocolon, and about the pancreas, (where they have been lately described by M. Lacauchie as organs connected with the lacteal vessels.) Imperfectly developed they may be found in the human fetus as early as the twenty-second week, and in fetal kittens.

The number of Pacinian corpuscles in an individual is very variable, and unaccountably so; it is hard to find them all, so as to reckon them up; but in man, from 150 to 350 have been counted on a single limb; and in the cat, from 50 to upwards of 200 in the omentum. In man, they are most numerous on the digital nerves, especially on the minute branches which are penetrating the cutis; and they may be found in abundance and very distinct on the nerves of the soles, as they run in the subcutaneous fat, and through it to the firmer layers of the cutis. Their arrangement, like their number, is uncertain; sometimes they are single and isolated; more often two or three are set together; sometimes they are placed in numerous groups.

Each Pacinian corpuscle is divisible into two parts: the corpuscle proper, and the stem by which it is attached to the nerve; and in each of these, as regards their structure, the nervous fibre and its investment have to be distinguished; for a single primitive nervous fibril runs through the stem of each corpuscle, and terminates at a little distance from the distal end, or peripheral pole of the corpuscle.

The corpuscles are elliptical, or ovate, or, more rarely, obovate: in man, they are often curved in their axis, so as to be semi-lunar or uniform, or even S-shaped. In man, the smallest (in a five-months-and-a-half fetus) measured ·08 to ·1 of a line in length, and ·032 to ·040 in breadth; in the adult the average size was from ·8 to 1·2 lines in length, and ·45 to ·6 in breadth; and once, one was found nearly two lines long. In external aspect they are uniformly semi-transparent, with a dull opal glistening, and an appearance of a white central cord. The stems are of various lengths and breadths; on an average they measure 1·5 by ·04 of a line. They go from the branch of the nerve at a right or acute, or more rarely, at an obtuse angle; and may be either straight or tortuous; and are inserted sometimes into the central or stem-end of the corpuscles, and sometimes into one or other side.

When the corpuscles are examined microspically they are sure to be marked with longitudinal lines, like meridian lines, of which the outermost are nearly parallel to the outer margin, the interior gradually more nearly parallel to the central axis line; the latter also are arranged much more closely than the former. These lines are the boundaries of so many distinct layers of the substance of which the greater part of the corpuscle is composed, as if by a series of from forty to sixty capsules enclosed one within the other. If a corpuscle be cut across transversely, these same layers appear as concentric rings, and on pressure, a small and variable quantity of clear fluid flows out from between them. The innermost of these capsules, of which the corpuscle may be said to be mainly composed, incloses a nearly cylindrical cavity, rounded, and a little dilated at the end which corresponds to the free or peripheral end of the corpuscle, and pointed at the other end. It is called the *central capsule*, and its *cavity*, which in man is, on an average, ·022 in diameter and ·4 in length, contains the nerve of the corpuscle and a fluid like that between the layers of the corpuscle, but, perhaps, a little more viscid. Next to this central capsule are a certain number of others which surround it very closely, have little or no fluid between them, and are sometimes so far distinct from those around them that they may be called the *system of internal capsules;* they comprise about half of the whole number of capsules. Occasionally, an appearance is seen as if partial transverse septa extended across the cavity between two or more adjacent capsules; and sometimes a dichotomous division of one of the meridian lines indicates either that a capsule has divided into two parts, or that parts of two capsules have been fused together. The several capsules hold together more tightly at the end of the corpuscle than anywhere else; and Pacini ascribed this to an *intercapsular ligament*, which he supposed to run from the surface of the end of the corpuscle to the end of its cavity, fastening the capsules together. But this is not apparent; their union at this part depends sometimes on their being set closer together with little or no fluid between them; sometimes, probably, on the existence of many of the septa before alluded to, and sometimes on the central cavity being curved or crook-shaped at its distal end so that the capsules following its form are, as it were, hooked one within the other. And another consequence of these varieties in the form of the distal part of the central cavity is, that as the capsules are always arranged in layers around it, the lines which mark their boundaries appear, in such cases, irregular and disarranged.

At the opposite, i. e. the proximal or stem end or pole of the corpuscle, the nerve enters. It is surrounded from the point at which it enters the corpuscle to the beginning of the central cavity by what is called the *conical process of the stem.* This is composed of very closely-arranged concentric layers, which appear to be the continuations of the several capsules of the corpuscle thus prolonged over the nerve; but about this there is a doubt: it is possible that at this part all the capsules have apertures into which the conical process of the stem inclosing the nerve is set.

The Pacinian corpuscles have blood-vessels which are distributed among the capsules, but do not penetrate so deeply as to reach those of the *internal system.*

Such is a description of the comparatively coarse anatomy of these corpuscles. Let us now follow the authors to that which is more minute, and in which we enter upon what belongs almost exclusively to the observations of Henle and Kölliker.

The fluid contained between the layers of the larger and fully developed corpuscules constitutes nearly three quarters of their whole mass; it is quite clear, with a slight tinge of yellow, and appears to be albuminous, like serum.

The capsules are composed of fibro-cellular tissue, with undulating fibrils, and round and elongated nuclei; and here and there the pale, straight, or branched filaments, insoluble in acetic acid. Each of the capsules (or, at least, of the outer ones,) is composed of a double layer of fibro-cellular tissue, an outer layer with transverse or circular fibres, and an inner layer with longitudinal fibres. The filaments insoluble in acetic acid are, for the most part, combined with the transverse fibro-cellular layer, and its fibres are also transversely arranged; the longitudinal fibres are imperfectly divided into fasciculi by elongated nuclei, which usually lie on the inner surface of the layers, and project into the intercapsular spaces. The outermost of the capsules is covered by loose fibro-cellular tissue, connecting it with the surrounding tissue, and very difficult to remove.

The most interesting part of the anatomy of these corpuscles is, of course, the nervous filament. A primitive nervous fibre enters through the stem into each corpuscle at one end, and after passing through its central cavity, terminates at the further end of the cavity, not with a loop, but either abruptly or with a bifurcated end, or with a more or less narrow curve;—*in every case the nervous fibril comes to an end.* The nerve of each corpuscle is derived from the branch on which the corpuscle is attached, and it passes rather tortuously through the corpuscle's stem and its conical process till it reaches the cavity of the corpuscle; in the stem it is closely surrounded by fibro-cellular tissue; in the corpuscle it lies free in the central cavity.

As far as the point at which it enters the cavity, the nervous fibril, going to a Pacinian corpuscle, differs in no respect whatever from the ordinary cerebro-spinal nerve-fibrils; and this is true as well of the fibrils belonging to the corpuscles in the mesentery, as of those in other parts. But when the fibril enters the central cavity, its form at once alters. The appearances which it presents in different aspects in this cavity indicate clearly that, on its entrance, the nervous fibril becomes paler, flattened, and smaller; and thus it continues, with some variations, and only very rarely assuming its previous form, throughout its course within the cavity of the corpuscle. On this fact of the nerve-fibril becoming smaller and flat within the cavity of the corpuscle, Henle and Kölliker raise the question whether a part only of the fibril may not be continued into the cavity; and the answer to this of course involves an answer to the question whether the substance of the nerve-fibrils be homogeneous or composed of an outer substance and an inner one (the axis-cylinder of Purkinje, the primitive band of Fontana and Remak.) At first they thought that that which passed on within the corpuscle was the axis-cylinder separated from what usually surrounds it, but they could not prove this; and the fact that, in some cases, the fibre regains its ordinary dark double-contoured appearance while within the corpuscle, contradicted the suspicion, and indicated that the change effected was only a diminution of size, and flattening of the fibril.

The great majority of the nerve fibrils terminate in the first corpuscles into which they enter: in a very few cases, the fibril passes right through one corpuscle into a second and terminates in it. In a very few cases, also, two fibrils are contained within one corpuscle. The most common mode in which the fibril terminates is in a knob-like swelling; the next most common is a more or less complete and deep dichotomous division. But as to the proportionate size of the terminal swelling, its particular form, the characters of its outlines and of its texture, whether uniform or variously granular, its distance from or closeness to the inner surface of the inmost capsule, in all these points there were so many varities that no general description can be given. The most careful examinations were made to detect whether the enlargement was a ganglion-corpuscle, but the result was against such an opinion.

Henle and Kölliker next enumerate several varieties which they have observed occasionally in the form and arrangement of the corpuscles and their contents; but, as we have already alluded to the chief of these, we shall pass on to the physiological portion of their work.

Of the mode in which the Pacinian corpuscles are developed, all that can be said is that they appear to be developed, like other normal structures, from cells, and acquire their peculiar form earlier than they do their proper textures; moreover, that the internal system of the capsules appears to be developed first, and the others appear to be formed from depositions around these; lastly, that the fluid begins to collect between the capsules when, in other respects, the formation of the corpuscles is nearly completed.

And now what use have these corpuscles? Cui bono? Cui malo? We may exclude the last, for they can hardly be thought the products or the causes of disease. For their inconstancy as to arrangement and mutual arrangement, their absence on most of the nerves, and their rare occurrences in certain animals—the only evidence for their being of pathological origin—are wholly insufficient to establish it; while their universal existence in the human subject, and in, at least, some animals, of whatever age or condition, their uniformly numerous occurrences in certain parts, and the uniformity and highly-organized character of their structures, are quite enough to prove them normal, physiological organs. And on these same grounds the opinion of Cruveilhier and Blandin, that they are accidental products, the consequence of pressure or some external circumstances, may be rejected.

Let us then have the opinion of Pacini himself. He believes that there is an analogy between these corpuscles and the electrical organs of the electric ray and other electric fish, like which these have the most essential structures of super-imposed layers separated by fluid. He says,

"While in the electric organ of the ray, both poles are placed externally, in our new organs one pole is external, the other is directed towards the nervous system or the centre of the animal sphere. As in a voltaic column, whose elements have the capsular form, or in a series of Leyden flasks which are inclosed one within the other, the one pole of the electric column would be placed in the interior, the other externally at the circumference of the apparatus, and a constant latent atmosphere of electricity would extend itself around the other. And although this electricity should not be actually evident, yet one might understand that, being exposed to the manifold influences of the vital principle, it might act as a conductor (as the other serves as a vehicle for the oscillations exerted by a luminous body,) to impart the mysterious manifestations of our will in the form of motions of some kind beyond the limits of our body, or to bring to our consciousness or observation external objects influencing us from without somewhat as in the disputed phenomena of animal magnetism." (p. 33.)

Pacini does not pretend to lay down principles for animal magnetism, nor to prove a connexion between it and the organs he has discovered; yet, having been deeply impressed with phenomena presented by persons whom he has seen in the magnetic somnambulism, he cannot help entertaining the hypothesis that these organs are "so many animal magnetomotors," supported as such an hypothesis is by strong analogies, "namely, on the one hand, the analogy between magnetism and animal electricity, and on the other, that between these organs and the electric apparatus of fish; and confirmed as it also is, by the obvious and remarkable agreement between the seats of the new organs and the mode in which the phenomena of animal magnetism are brought about."

It need not surprise us, as Henle and Kölliker say, if the animal magnetizers greedily lay hold on such a discovery as this, the first anatomical fact, we will venture to say, that was ever found unopposed to their notions; but we beg them next to try their passes near a cat's belly (of course without gloves,) for the abundance of Pacinian corpuscles thereabouts should produce some wonderful event.

However, if we reject this mesmeric hypothesis, we must confess we have no other to propose. Henle and Kölliker think that that which is most probable is that these organs *are* allied to the electric organs of fish. They certainly have nothing to do with motion, for they occur on no motor nerves; nor can they be connected with acuteness of sensation, for they are abundant on sympathetic nerves, through which no ordinary impressions produce sensation, and they are not found on some nerves that are peculiarly sensitive, e. g. the glosso-pharyngeal and the fifth. They have also the alternate layers of membranes and fluid, like electric organs; yet the experiments which our authors made to detect free electricity in them (in the mesenteries of live cats) yielded no distinct evidence of its existence. With them, therefore, we leave others to settle this question.

